# Use of multiple data sources to estimate hepatitis C seroprevalence among prisoners: A retrospective cohort study

**DOI:** 10.1371/journal.pone.0180646

**Published:** 2017-07-07

**Authors:** Kathryn J. Snow, Alun H. Richards, Stuart A. Kinner

**Affiliations:** 1 Melbourne School of Population and Global Health, University of Melbourne, Melbourne, Victoria, Australia; 2 Centre for International Child Health, University of Melbourne Department of Paediatrics and Murdoch Childrens Research Institute, Royal Children’s Hospital, Melbourne, Australia; 3 Communicable Diseases Branch, Prevention Division, Department of Health, Queensland Government, Brisbane, Queensland, Australia; 4 Griffith Criminology Institute, Griffith University, Brisbane, Queensland, Australia; 5 Mater Research Institute, University of Queensland, Brisbane, Queensland, Australia; 6 School of Public Health and Preventive Medicine, Monash University, Melbourne, Victoria, Australia; 7 Centre for Adolescent Health, Murdoch Childrens Research Institute, Melbourne, Victoria, Australia; University of Queensland, AUSTRALIA

## Abstract

Hepatitis C is a major cause of preventable morbidity and mortality. Prisoners are a key population for hepatitis C control programs, and with the advent of highly effective therapies, prisons are increasingly important sites for hepatitis C diagnosis and treatment. Accurate estimates of hepatitis C prevalence among prisoners are needed in order to plan and resource service provision, however many prevalence estimates are based on surveys compromised by limited and potentially biased participation. We aimed to compare estimates derived from three different data sources, and to assess whether the use of self-report as a supplementary data source may help researchers assess the risk of selection bias. We used three data sources to estimate the prevalence of hepatitis C antibodies in a large cohort of Australian prisoners–prison medical records, self-reported status during a face-to-face interview prior to release from prison, and data from a statewide notifiable conditions surveillance system. Of 1,315 participants, 33.8% had at least one indicator of hepatitis C seropositivity, however less than one third of these (9.5% of the entire cohort) were identified by all three data sources. Among participants of known status, self-report had a sensitivity of 80.1% and a positive predictive value of 97.8%. Any one data source used in isolation would have under-estimated the prevalence of hepatitis C in this cohort. Using multiple data sources in studies of hepatitis C seroprevalence among prisoners may improve case detection and help researchers assess the risk of selection bias due to non-participation in serological testing.

## Introduction

Compared to the general public, the prevalence of hepatitis C and other blood borne viruses is elevated among prisoners around the world[1]. Left untreated, a substantial proportion of hepatitis C infections progress to liver cirrhosis, and then to potentially fatal liver cancer or liver failure[2]. Highly effective therapies are now available, and prisoners are a key target population for hepatitis C control programs[3].

Even outside prisons, hepatitis C is a common disease, affecting an estimated 1–2% of the general population in high-income countries[4]. Given the high drug costs associated with new treatments, estimates of the total cost and relative cost-effectiveness of programs targeting particular key populations may be sensitive to small changes in prevalence estimates[5]. In planning prison-based treatment programs and estimating their potential impacts on national hepatitis C epidemics, accurate estimates of the prevalence of hepatitis C among prisoners are critical.

Both hepatitis C serosurveys and routine testing in prisons often achieve limited participation, in the range of 40–80% of those eligible to participate[6–8]. Participation even at the upper limit of this range introduces risk of selection bias, particularly when participation is plausibly related to a participant’s risk of having the condition studied. For example, people who are aware of their positive status may decline testing because they consider it redundant or for fear of stigmatisation, while people who have never injected drugs may decline because they believe they are not at risk. With common conditions such as hepatitis C, the impact of even moderate differences in risk between participants and abstainers may have material impacts on prevalence estimates, and thus on responses premised on those prevalence estimates. As such, although many estimates of the prevalence of HCV antibodies among prisoners have been published, some may be inaccurate.

The use of multiple data sources might improve hepatitis C case detection in prison-based studies, and help researchers assess the risk of selection bias compared to using estimates from any one approach. The aim of this study was to assess the concordance between three sources of data on HCV seropositivity in an incarcerated cohort (records of positive tests in prison, self-reported status, and notifiable conditions surveillance data regarding positive tests), and to ascertain the potential for multiple data sources to improve estimates of hepatitis C prevalence in prisons.

## Methods

### Hepatitis C prevalence in the cohort

The Passports cohort includes 1,325 adults recruited in the weeks prior to release from the seven largest prisons in the Australian state of Queensland between August 2008 and July 2010. Women were deliberately oversampled to allow sex-stratified analyses (25% of the cohort were women, compared to 11% of released prisoners[9]). In other regards, the cohort was demographically similar to people released from prison in the state during the recruitment period[10]. Eligibility screening and recruitment was carried out within prisons, by research staff independent of the prison authorities. Of those approached and found to be eligible, 80% agreed to participate[10].

The Passports study was approved by The University of Queensland’s Behavioural and Social Sciences Ethical Review Committee. Approval for linkage to data from the Queensland Notifiable Conditions System was provided by the Queensland Health Human Research Ethics Committee and under the *QLD Public Health Act (2005)*. All participants provided written, informed consent to participate.

Participants completed a detailed baseline interview on a variety of health-related topics, including their history of testing for blood borne viruses and the results of their most recent tests (Q1: “Have you ever been tested for hepatitis C?” A: “No”; “Yes, in the last year”; “Yes, more than one year ago”; “Unsure / can’t remember”. Q2: “If yes, what was the result of your last hepatitis C test?” A: “Don’t have hepatitis C (negative)”; “Have hepatitis C (positive)”; “Don’t know / didn’t get result”). Trained research staff independent of both the state prison health service and the correctional authorities conducted the interviews. Participants were asked to consent to a review of their prison medical records, and to data linkage with state health records including the State’s notifiable diseases surveillance system. Health record linkage was carried out probabilistically using participant name and all known aliases, date of birth, postcodes of residence, and gender, in accordance with a previously validated method that has been shown to have a sensitivity and specificity of 99.9% [11].

In-prison medical records covered the period prior to the interview date with no left censoring (the earliest record was from September 1993), however records pertaining to previous incarceration episodes were omitted from this analysis as the risk of seroconversion between incarceration episodes is considerable[12]. State health records used in this analysis included reports made to the Queensland Notifiable Conditions Surveillance System (hereafter “surveillance records”) for the period November 1997 to the interview date. Hepatitis C notification is mandated in Queensland, with reports required by clinicians and laboratories upon either a positive serological test, or a positive test for viral nucleic acid[13]. State health records for 10 participants were not linked successfully (7 participants did not consent to record linkage, and 3 were not linked due a computer error during the linkage process). These participants were excluded from this analysis, giving a final sample of 1,315 individuals (1,037 men and 278 women).

The surveillance records used in this analysis distinguish between positive results on serological and nucleic acid method tests—either was counted as an indicator of hepatitis C seropositivity. The data extracted from prison medical records denotes simply a positive test for hepatitis C, without distinguishing between seropositivity and active infection. Likewise, our interview tool did not distinguish between antibody and nucleic acid testing, as this distinction is poorly understood by many Australian primary care providers[14] and by many Australians at risk of or living with HCV infection[15]. As such, we refer to “hepatitis C seropositivity” throughout, although as Australia was a setting of low treatment uptake before and during our study period, it can be assumed that around 70–80% of our seropositive participants would have been living with chronic hepatitis C infection[2].

### Data analysis

We calculated the following proportions: the proportion of the cohort tested by each measure, the proportion of the cohort identified as seropositive by each measure, and the proportion of those tested who tested positive. Negative tests are not recorded by the surveillance system, which precluded calculating the proportion tested and the proportion of those tested who tested positive for this measure. We calculated the proportion of the cohort with no indication of HCV status according to any of the three data sources. A Venn diagram was produced to illustrate the extent of overlap between the three sources with regard to the number of participants identified as seropositive using the open access web application EulerAPE.

We calculated the sensitivity, specificity, positive and negative predictive value of self-report against the results of tests in prison, supplemented with positive results from the surveillance data. This analysis was restricted to 570 participants of known status who gave a definitive answer to the interview question about their test results. Reference group negatives were defined as those with a negative test in prison and no conflicting surveillance record; reference group positives were defined by either a positive test in prison or a surveillance record. We also calculated the sensitivity of in-prison testing against self-report and against surveillance records.

## Results

### Hepatitis C seroprevalence in the cohort

The cohort comprised 1,315 participants with a median age of 30 years. Demographic and other data are summarized in Table 1. A total of 445 participants (33.8% of the cohort) had at least one indicator of hepatitis C seropositivity. Table 2 details the numbers and proportions of participants who tested positive according to each data source, and all data sources. Hepatitis C status was unavailable from any of the three data sources for 12.1% of the cohort; these participants either did not know or who chose not to disclose their status at interview, in addition to having no record of a test in prison and no surveillance record.

**Table 1:** Baseline characteristics of the cohort.

Characteristc	Value
Male (%)	78.9
Age in years (median, IQR)	30 (24, 38)
First custodial sentence (%)	33.7
Time served, months (median, IQR)	6 (3, 12)
Lifetime history of IDU, self-reported (%)	58.9
Age in years at initiation of injecting (median, IQR)	17 (15, 20)

**Table 2:** Proportions of participants identified as HCV seropositive according to each individual data source and all three sources combined, among cohort and among those tested.

Measure	Number (% cohort) tested	Number positive	% of cohort HCV+ (95% CI)	% of those tested HCV+ (95% CI)
Prison test	672 (51.1%)	194	14.8 (12.9, 16.8)	28.9 (25.5, 32.5)
Self-report	1,164 (88.5%)	356	27.1 (24.7, 29.6)	30.6 (27.9, 33.3)
Surveillance	-	327	24.9 (22.6, 27.3)	-
Any source	-	445	33.8 (31.3, 36.5)	-

### Discordance between data sources

**Fig 1:**
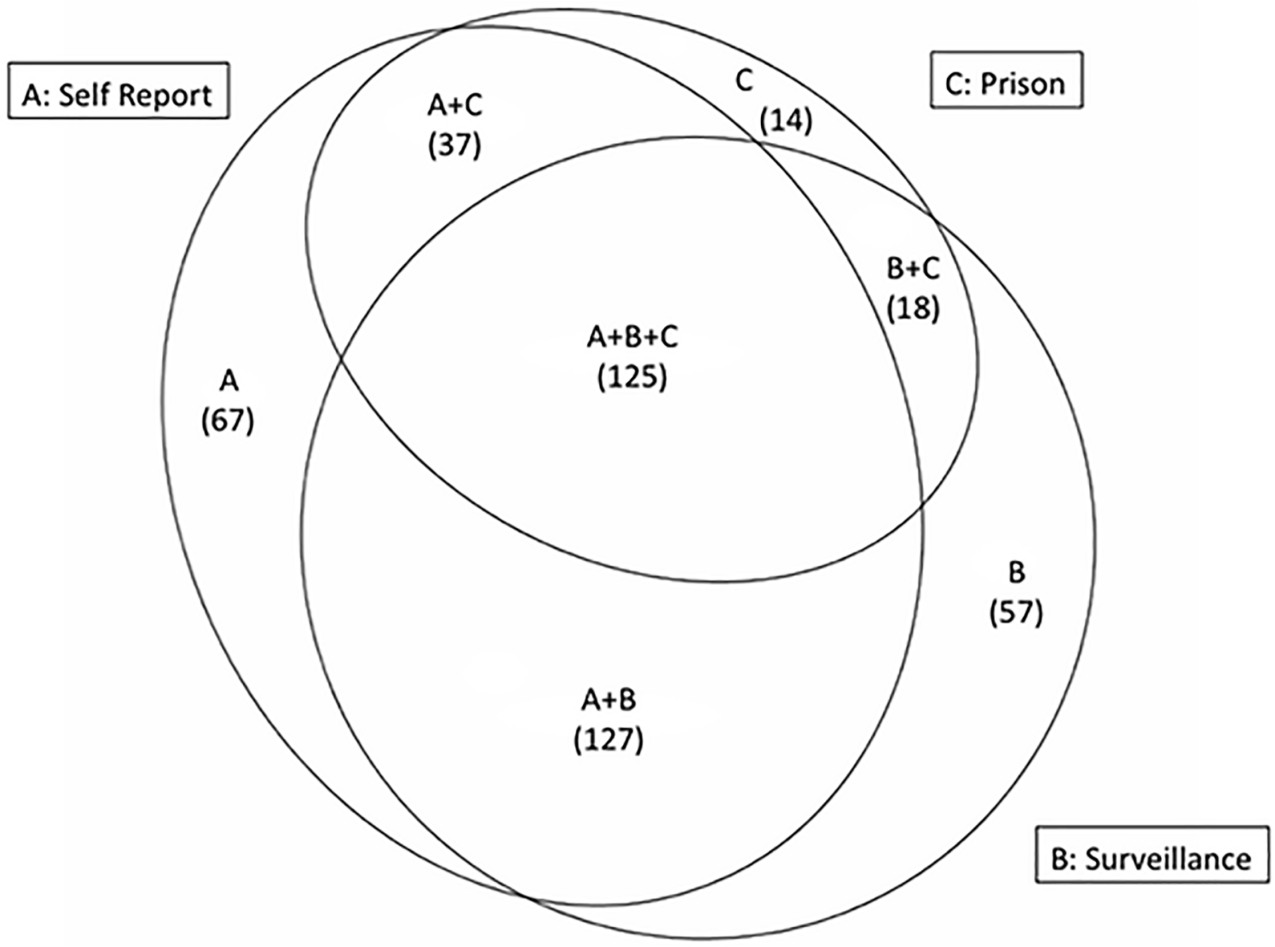
Overlap between each data source regarding HCV seropositive participants (A: self-report. B: surveillance records. C: In prison testing. Figure to scale.).

In addition to discrepancies in the seroprevalence estimates given by each data source, there were considerable discordances between each data source with regard to which individual participants were identified as being HCV seropositive (Fig 1). Overlap between data sources was highest between surveillance records and self-report, intermediate between self-report and prison medical records, and lowest between surveillance records and prison medical records. Only 28.4% of those with any indication of infection appeared in all three data sources (Fig 1: A+B+C), however most participants appeared in at least two data sources. Prison testing had identified 43.7% of those who had a surveillance record for HCV prior to the baseline interview (143 of 327), and 45.5% of those who self-reported a history of infection (162 of 356).

### Validation of self-report

A total of 570 participants had biomedical evidence of HCV serostatus and gave a definitive answer to the interview question about their test results. A large majority of patients who self-reported being HCV seropositive had either a positive test in prison, or a surveillance record, however 22.8% of those who stated that they were HCV-negative at interview had either tested positive during their current incarceration episode, or had a surveillance record. Compared against in-prison tests and surveillance records, the sensitivity of self-report was 80.1%, the specificity was 97.4%, the positive predictive value was 97.8%, and the negative predictive value was 77.2%.

## Discussion

This study investigated the apparent seroprevalence of hepatitis C in an incarcerated cohort according to three different measures–self-reported history of infection during face-to-face interview, state-based notifiable conditions surveillance records, and prison medical records. We observed substantial discordance between data sources, with a low proportion of apparently HCV seropositive participants identified through testing in prison. Self-report had very high specificity and positive predictive value against biomedical indicators. Our findings suggest that any one method used in isolation will underestimate the true prevalence of hepatitis C among prisoners, and that self-report may be a useful supplementary source of information in prison-based HCV serosurveys.

The seroprevalence estimate of 33.8% given by our pooled data sources is somewhat higher than the prevalence among those who accepted testing in prison (28.9%), and more than twice the percentage of the cohort actually identified through in-prison testing alone (14.8%). Given the substantial proportion of the cohort with no documented history of testing and no reported knowledge of their status (12.1%), the true prevalence of HCV antibodies in our cohort may be higher still. It should be noted that as hepatitis C is more common among incarcerated women than incarcerated men in Australia[16], the prevalence in this cohort is likely to be slightly higher than among the general prison population in our state, owing to the intentional oversampling of women in this study.

A previous review of HCV surveillance in our setting found evidence of substantial redundant serological testing among people who had previously tested positive[17]. Such tests are a waste of resources and of clinician time, an unnecessary medical procedure for prisoners who may have poor vein health, and they may delay more clinically useful tests for current infection status and HCV genotype. Surveillance data may be an under-utilised resource in many settings, particularly when held by the same authorities (e.g. state departments of health) that administer prison health services. Services such as the Australian cervical cytology registries provide one model of programs that combine disease surveillance with data sharing and decision support for clinicians; such models are increasingly relevant for viral hepatitis, particularly as treatment uptake for hepatitis C increases with new therapies[18]. As large numbers of patients begin to access care for hepatitis C both in prisons and in the community, systems to enable health care providers to track engagement with care (with patient consent) will require serious consideration.

One finding of concern here is that a substantial number of participants who tested positive in prison were not identified in the surveillance data, suggesting that some positive tests may not have been reported to state health authorities, a documented issue in hepatitis surveillance in Australia[19]. Some surveillance records may have been missed during the linkage process, although the method used here has been validated previously against administrative health data sets, and was found to have an error rate of less than 0.1%[11]. It is possible that in this study the accuracy may have been lower, given that the use of aliases in this cohort was relatively common and that the condition under consideration is a sensitive one. If this were the case, however, it would not alter the substantive findings of this study–that any single data source would have under ascertained HCV seropositivity, and that the positive predictive value of self-report was very high.

It is notable that although past studies have cast doubt on the reliability of self-report with regard to stigmatised topics[20,21], our participants disclosed a variety of illegal and stigmatised experiences during interviews (e.g. injecting drugs in prison, histories of sexually transmitted infections)[22], suggesting that in general their responses were candid and honest. The moderate sensitivity and very high positive predictive value of self-report (80.1% and 97.8% respectively) observed in this study suggests that self-report is a potentially useful supplementary source of data on HCV seropositivity in this population. This is consistent with previous findings in Australia[23] and the United States[24]. Self-report identified substantially more HCV seropositive participants than routine testing did, and in-prison tests or surveillance records corroborated an overwhelming majority of self-reported HCV infections.

A proportion (22.8%) of participants with evidence of HCV seropositivity reported at interview that the result of their last test was negative. There are several potential explanations for this. Some participants may have simply chosen not to disclose a positive test result, or they may not have been aware of their positive status after recent seroconversion (for example if they had not attended a follow-up appointment to receive their most recent test results). However, some participants may have been accurately reporting a negative test for HCV RNA indicating either successful treatment or spontaneous clearance. If this were the case, then the negative predictive value of self-reported status would be higher than we have reported here.

Our findings suggest that although some people with HCV antibodies may choose not to disclose their status during research interviews, those participants who do choose to disclose a history of HCV infection are highly likely to be correct about their status. Individuals who know themselves to have a history of infection, or who have recently tested negative, may decline testing in both routine care and in research.

In HCV serosurveys where some participants do decline testing, self-report may be a useful tool for investigators hoping to assess the risks of selection bias. If the self-reported prevalence of HCV antibodies among those who decline testing is markedly different than the observed prevalence among those who accept testing, researchers have good reason to believe that selection bias may have occurred. If the prevalence according to self-report among those who decline testing is higher than was observed among those who are tested, it should be considered likely the true prevalence in the source population has been underestimated.

The substantive limitation of this study is that we did not offer hepatitis C testing to our participants, and as such did not have a ‘gold standard’ through which to ascertain true negative status in those participants who had not been tested in prison during their current sentence. However, it is likely that if we had offered testing, we would have faced the same low participation that has limited other studies. The largest regular survey of blood borne viruses among prisoners in Australia, the triennial National Prison Entrants Blood Borne Virus and Risk Behaviour Survey, achieved an overall response fraction of 82% in Queensland (the setting of our study), with 282 participants. However of these, only 128 (45% of participants, and 37% of those eligible) accepted hepatitis C testing [16]. Similarly, our study achieved a response fraction of 80% at the recruitment stage[10], and among the participants, 53% had a record of a HCV test in prison (42% of those eligible to participate).

A major strength of this study was its utilisation of multiple data sources that are not usually available in prison health research, particularly the linked notifiable conditions surveillance data. When these sources were combined, there was at least one source of data available on HCV serostatus for 87.9% of our participants.

A second limitation is the time that has elapsed since our participants were recruited in 2008–2010. It is possible that acceptance of routine testing in prisons in our study setting has changed since our study began, and our findings should be interpreted with this in mind.

### Conclusions

Prisoners are a key population for hepatitis C, and the management of hepatitis C among prisoners should form a major part of any national plan for hepatitis C control[3,25]. Planning appropriately for service provision and estimating the potential impact of expanded treatment programs on national hepatitis C burden requires accurate prevalence estimates, and high case detection by prison health services. The reliability of prevalence estimates derived through serosurveys or routine screening in prisons is limited by low participation, and routine testing may substantially under-ascertain the burden of hepatitis C among prisoners.

Our findings suggest that prison medical records, notifiable conditions surveillance data, and self-reported status may each be valuable and complementary data sources in prison-based surveys of HCV seroprevalence. With consent, cases identified through prison medical records or surveillance data could be added to the numerator and denominator of prevalence estimates, supplementing data from those participants who do consent to serological testing during surveys. The very high positive predictive value of self-report observed here suggests that self-report may be a useful indicator of the risk of selection bias in surveys with limited participation.

## Supporting information

S1 File Aggregate data on hepatitis C seroprevalence within the passports cohort. (XLSX)
